# Current News

**Published:** 1905-10

**Authors:** 


### Current News.

### MISSISSIPPI DENTAL ASSOCIATION.

The twelfth annual meeting of the Mississippi Dental Association, held in Jackson, April 19 to 21, 1905, elected the following officers:

President, Dr. A. B. Kelly, Yazoo City; First Vice-President, Dr. L. B. McLaren, Natchez; Second Vice-President, Dr. J. F. Scott, Summit; Secretary, Dr. E. Douglas Hood, Tupelo; Corresponding Secretary, Dr. W. H. Reaben, McComb City; Treasurer, Dr. C. C. Crowder, Kosciusko.

*Executive Committee.*—Dr. W. O. Talbot, Biloxi; Dr. C. F. Boger, Natchez; Dr. E. Douglas Hood, Tupelo, Chairman, *ex officio.*

The next meeting promises to be the best ever held, and preparations are now being made for a good series of papers and some interesting clinics.

The thirteenth annual meeting will be held in Gulfport, between the 1st and 15th of June, 1906. Exact date to be fixed by the Executive Committee.

E. Douglas Hood,
*Secretary.*

### OFFICERS OF THE NATIONAL ASSOCIATION OF DENTAL EXAMINERS.

President, H. W. Campbell, D.D.S., Suffolk, Va.; Vice-President from the West, F. O. Hetrick, D.D.S., Ottawa, Kan.; Vice-President from the South, F. A. Shotwell, D.D.S., Rogersville, Tenn.; Vice-President from the East, George E. Mitchell, D.D.S., Haverhill, Mass.; Secretary and Treasurer, Charles A. Meeker, D.D.S., Newark, N. J.

*Committee on Colleges.*—J. G. Reid, D.D.S., *Chairman*, Chicago, Ill.; George E. Mitchell, D.D.S., Haverhill, Mass.; J. J. Wright, D.D.S., Milwaukee, Wis.

*Committee on Conference.*—J. F. Dowsley, D.D.S., *Chairman*, Boston, Mass.; F. O. Hetrick, D.D.S., Ottawa, Kan.; R. H. Walker, D.D.S., Norfolk, Va.

*Membership Committee.*—M. F. Finley, D.D.S., *Chairman*, Washington, D. C.; Thomas Cole, D.D.S., Newman, Ga.; C. R. Taylor, D.D.S., Streator, Ill.

*State Advisory Committee.*—Henry Barnes, M.D., Cleveland, Ohio; George E. Mitchell, D.D.S., Haverhill, Mass.; E. P. Dameron, D.D.S., St. Louis, Mo.; C. H. Oakman, D.D.S., Detroit, Mich.; W. G. Mason, D.D.S., Tampa, Fla.

*Committee for Promoting Relations with Foreign Examiners.*—T. J. Barrett, D.D.S., *Chairman*, Worcester, Mass.; F. A. Shotwell, D.D.S., Rogersville, Tenn.; F. C. James, D.D.S., Winona, Minn.; C. Stanley Smith, D.D.S., Cincinnati, Ohio.

*Committee on Resolutions.*—H. C. Brown, D.D.S., Columbus, Ohio; C. S. Stockton, D.D.S., Newark, N. J.; F. F. Drew, D.D.S., Baltimore, Md.

*Committee on Contracts.*—Charles A. Meeker, D.D.S., Newark, N. J.

*Committee on Tabulation of Examiners' Reports of Examinations.*—Alphonso Irwin, D.D.S., Camden, N. J.

---

### MISSISSIPPI VALLEY MEDICAL ASSOCIATION.

At the next meeting of the Mississippi Valley Medical Association, to be held at Indianapolis, Ind., October 10, 11, 12, 1905, the annual addresses will be delivered by Dr. Arthur R. Edwards, of Chicago, and Dr. W. D. Haggard, of Nashville, Tenn.

Dr. Edwards has chosen for the subject of his address, "Certain Phases of Uræmia: Their Diagnosis and Treatment," and Dr. Haggard will discuss, in his address, "The Present Status of Surgery of the Stomach." In addition to these addresses there will be the annual address of the President, Dr. Bransford Lewis, of St. Louis.

A cordial invitation is extended to every physician in the valley to attend this meeting, for which a large number of interesting and valuable papers have been promised.

Henry E. Tuley, M.D.,
*Secretary.*

---

### NATIONAL ASSOCIATION OF DENTAL FACULTIES.

The twenty-second annual meeting of the National Association of Dental Faculties, held at Buffalo, N. Y., July 27 and 28, 1905, resulted in the election of the following officers and committees:

President, J. H. Kennerly, 2645 Locust Street, St. Louis, Mo.; Vice-President, J. I. Hart, New York; Secretary, George Edwin Hunt, 131 East Ohio Street, Indianapolis; Treasurer, H. R. Jewett, Atlanta, Ga.

*Executive Committee.*—D. J. McMillan, Kansas City; L. P. Bethel, Columbus, Ohio; J. B. Wilmot, Toronto; R. M. Sanger, East Orange, N. J.; H. B. Tileston, Louisville.

*Ad Interim Committee.*—S. H. Guilford, Philadelphia; M. C. Marshall, St. Louis; J. P. Gray, Nashville.

*Foreign Relations Committee.*—G. V. Black, Chicago; W. F. Litch, Philadelphia; D. R. Stubblefield, Nashville; William Carr, New York; J. D. Patterson, Kansas City.

Forty-three of the fifty colleges holding membership were represented by delegates, and a most harmonious meeting was held. United States Consul J. H. Worman, Munich, Germany, was present at one session, and told what was being done to rehabilitate the American degree in that country. Announcement was also made that the United States government had recognized the National Association of Dental Faculties in its act regulating the practice of dentistry in the Philippine Islands.

George E. Hunt,
*Secretary.*

